# The impact of general anesthesia on the outcomes of preterm infants with gestational age less than 32 weeks delivered via cesarean section

**DOI:** 10.3389/fphar.2024.1360691

**Published:** 2024-03-20

**Authors:** Lijun Wang, Chengxiao Liu, Xiaokang Wang, Sha Zhu, Ligong Zhang, Bo Wang, Yonghui Yu

**Affiliations:** ^1^ Department of Neonatology, Shandong Provincial Hospital Affiliated to Shandong First Medical University, Jinan, China; ^2^ Department of Anesthesiology, Shandong Provincial Hospital Affiliated to Shandong First Medical University, Jinan, China

**Keywords:** cesarean section, very preterm infants, general anesthesia, neuraxial anesthesia, neonatal outcomes

## Abstract

**Background:**

Recent advancements in China’s perinatal and neonatal intensive care have significantly reduced neonatal mortality, yet preterm births before 32 weeks remain the primary cause of neonatal fatalities and contribute to long-term disabilities. The prognosis of very preterm infants (VPIs) is significantly affected by factors including the intrauterine environment, delivery method and neonatal intensive care. Cesarean section which often used for preterm births has implications that are not fully understood, particularly concerning the type of anesthesia used. This study examines the impact of general anesthesia (GA) during cesarean delivery on VPI outcomes, aiming to identify strategies for mitigating GA-associated risks.

**Methods:**

This cohort study analyzed 1,029 VPIs born via cesarean section under 32 weeks’ gestation at our single-center from 1 January 2018, to 31 December 2022. Detailed medical records, encompassing perioperative information, maternal data and neonatal outcomes were meticulously examined. The primary aim of this investigation was to compare maternal characteristics and neonatal outcomes between VPIs delivered under GA and neuraxial anesthesia (NA). A significance level of *p* < 0.05 was established.

**Results:**

Of the 1,029 VPIs analyzed, 87.95% (n = 905) were delivered via NA and 12.05% (n = 124) via GA. Mothers with hypertensive pregnancy diseases and emergency operations were more inclined to choose GA. VPIs delivered under GA showed a lower Apgar score at one and 5 minutes (*p* < 0.01), increased need for tracheal intubation resuscitation (32.2% vs. 12.2%, *p* < 0.01) and a greater incidence of severe neurological injury (SNI) (14.5% vs. 5%, *p* < 0.01). Multivariable analysis revealed GA was significantly associated with lower Apgar scores at one (OR 6.321, 95% CI 3.729–10.714; *p* < 0.01) and 5 minutes (OR 4.535, 95% CI 2.975–6.913; *p* < 0.01), higher risk of tracheal intubation resuscitation (OR = 3.133, 95% CI = 1.939–5.061; *p* < 0.01) and SNI (OR = 3.019, 95% CI = 1.615–5.643; *p* < 0.01). Furthermore, for VPIs delivered under GA, a prolonged interval from skin incision to fetus delivery was associated with a lower 5-min Apgar score (*p* < 0.01).

**Conclusion:**

This study revealed the significant impact of GA on adverse outcomes among VPIs. In cases when GA is required, proactive measures should be instituted for the care of VPIs such as expediting the interval from skin incision to fetal delivery.

## Introduction

Various factors exert influence on the prognostic outcomes of preterm infants, among which the mode of delivery holds particular significance. Recent studies have demonstrated that cesarean sections performed for standard obstetrical indications are linked to improved morbidity and mortality among preterm infants. (Holzer et al., 2016; Karayel Eroglu et al., 2022). Hence, it is noteworthy that preterm infants, particularly very preterm infants (VPIs), are frequently delivered via cesarean section. There exists a well-established global consensus favoring the use of neuraxial anesthesia (NA) techniques (such as spinal anesthesia, epidural anesthesia, or combined spinal and epidural anesthesia) for cesarean sections. This preference is underpinned by compelling evidence demonstrating improved maternal outcomes (Mushambi et al., 2015; American Society of Anesthesiologists Committee on Standards and Practice Parameters., 2016), including reduced risks of short-term maternal morbidity or mortality, decreased incidence of wound infections, and alleviated postoperative pain (Chattopadhyay et al., 2014; Sayed et al., 2015).

General anesthesia (GA) stands out for its ability to provide the shortest onset time, making it a viable choice in cases of urgent medical or obstetric indications necessitating cesarean section (Palanisamy et al., 2011; Butwick et al., 2015). However, for preterm infants delivered via cesarean section, the criteria determining the selection of GA assume critical clinical significance. Anesthesiologists must carefully weigh these determinants while prioritizing the safety of both the maternal and neonates. Several studies have demonstrated that the GA group exhibits lower newborn Apgar scores and higher rates of neonatal intensive care unit (NICU) admissions compared with the NA group (Bao et al., 2022; Shi et al., 2024). While some research has explored maternal and neonatal outcomes associated with GA administration during cesarean sections (Nwafor et al., 2014; Butwick et al., 2015), there remains a notable lack of studies specifically addressing the impact of GA on the outcomes of VPIs delivered by cesarean section.

Due to the rising incidence of preterm births, SNI has emerged as a progressively concerning complication that significantly influences the prognoses of preterm infants. Correctly managing the processes of delivery and neonatal resuscitation assumes paramount importance as crucial steps in mitigating the adverse outcomes associated with SNI in preterm infants. Our research endeavors have encompassed a series of cohort studies, leveraging comprehensive databases to assess the myriad factors influencing the outcomes of VPIs. However, one critical facet of our investigations has hitherto remained unexplored: the impact of anesthesia modes on the outcomes of VPIs delivered via cesarean section. In the current study, we have undertaken an investigation to ascertain whether distinct anesthesia modalities (GA or NA) exhibit associations with neonatal adverse outcomes in VPIs.

## Methods

### Study design and setting

**FIGURE 1:**
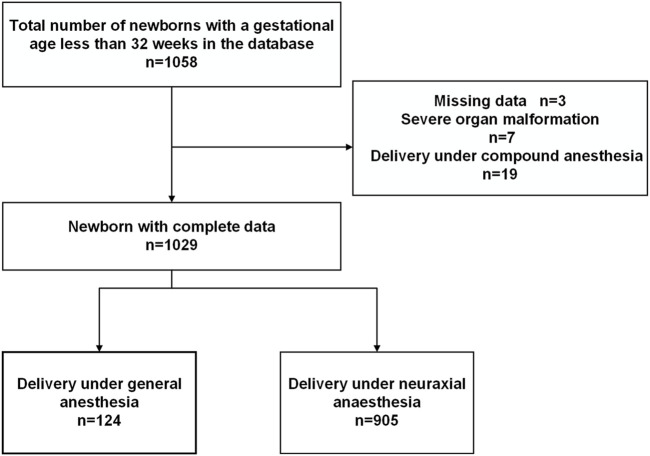
Flow diagram for newborn selection.

The study cohort utilized in our research was derived from a dataset established through the Sino-northern Neonatal Network (SNN), a network characterized by ongoing prospective data collection. A comprehensive description of this network and the methodologies employed for the assessment of post-natal conditions and the quality control of resuscitation procedures in preterm infants has been previously documented in other publication (Dong et al., 2023). We conducted data analysis on a cohort comprising 1,058 preterm infants with a gestational age of less than 32 weeks who were delivered via cesarean section. This cohort was drawn from cases occurring between 1 January 2018, and 31 December 2022, at our singular tertiary care institution (Shandong Provincial Hospital Affiliated to Shandong First Medical University, China). The analysis encompassed maternal demographic information and the subsequent comparison of neonatal outcomes. The choice of anesthesia method was determined by the attending anesthesiologist, taking into consideration the urgency of the surgical procedure and maternal complications. All aspects of maternal anesthesia management adhered to established practice guidelines for obstetric anesthesia, as outlined by the American Society of Anesthesiologists Committee on Standards and Practice Parameters (American Society of Anesthesiologists Task Force on Obstetric Anesthesia and the Society for Obstetric Anesthesia and Perinatology, 2016). Infants with documented severe congenital malformations, those with partial data availability, or those delivered under compound anesthesia were excluded from the study. Ultimately, a total of 1,029 eligible VPIs were included in this investigation (Figure 1).

### Definition of variables

Severe neurological injury (SNI) was defined as the presence of intraventricular hemorrhage (IVH) of grade 3 or 4 and/or porencephalic ventricular leukomalacia (PVL) (Egesa et al., 2021). To detect cerebral lesions, the standard protocol involved conducting one or two cranial ultrasound scans within the first 48 h post-birth, followed by weekly scans for the subsequent 2 weeks. In instances where cranial ultrasound is unfeasible, magnetic resonance imaging (MRI) is utilized, either at 36 weeks’ postmenstrual age (PMA) or upon discharge. We categorized bronchopulmonary dysplasia (BPD) as necessitating respiratory support at 36 weeks’ PMA (Strueby and Thébaud, 2018). Severe retinopathy of prematurity (ROP) was identified as ROP stage 3, 4, or 5, and/or cases receiving ROP treatment (laser or intraocular injection) (Chiang et al., 2021). Severe necrotizing enterocolitis of newborn (NEC) was defined as NEC stage 2 or 3, following Bell’s criteria (Kim, 2014). The Score for Neonatal Acute Physiology, Version II (SNAP-II), a standardized and clinically validated tool, is employed to assess the severity of newborn illnesses and mortality risk, with scoring criteria based on prior studies (Richardson et al., 2001).

### Statistical analysis

SPSS 26.0 statistical software was used for analysis. Normally distributed continuous variables are expressed as means ± standard deviation, abnormally distributed continuous variables are expressed as median (interquartile range) and categorical variables are expressed as number (percentage). Quantitative indicators are compared by *t*-test or Wilcoxon rank-sum test according to the data distribution, while categorical indicators are compared by chi-square test or Fisher’s exact probability method (if chi-square test is not applicable). Regarding neonatal outcomes, variables significantly correlated with the mode of anesthesia in univariate analyses were retained as covariates in a multivariable logistic regression model. The model’s goodness of fit was evaluated using the Hosmer–Lemeshow statistic, and a *p*-value < 0.05 was considered statistically significant while confidence intervals (CIs) were set at 95%.

## Results

### Medical and obstetrical indications for parturients receiving general anesthesia

**FIGURE 2:**
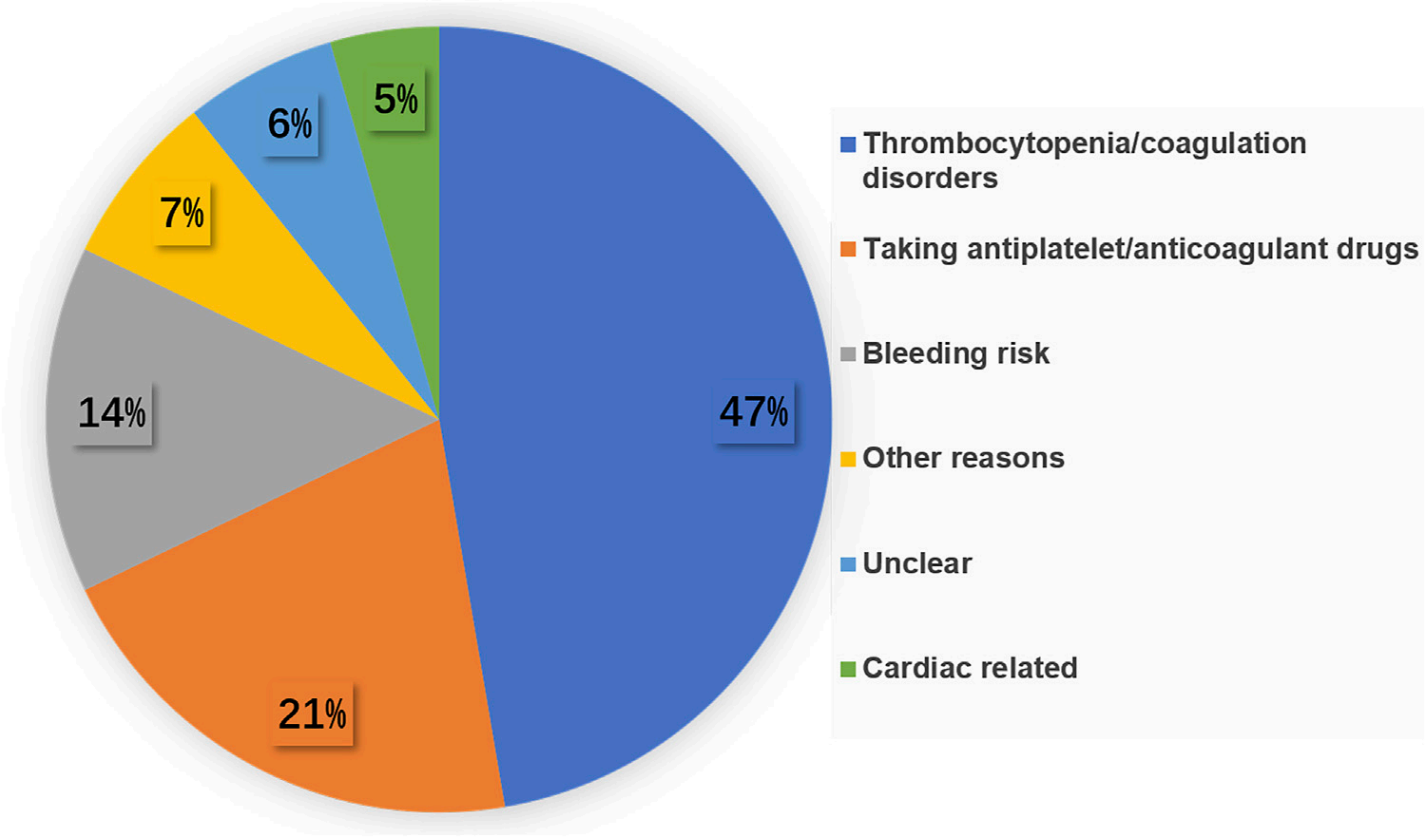
Medical and obstetrical factors to undergo general anesthesia during cesarean section.

Our study included 1,029 VPIs delivered via cesarean section before 32 weeks of gestational age. Of these, 124 were delivered under GA and 905 under NA. Due to multiple birth, 124 VPIs in GA group originated from 112 parturients. The indications for GA included: maternal thrombocytopenia or coagulation disorders (47%), taking antiplatelet/anticoagulation drugs (21%), bleeding risk (14%), cardiac related diseases (5%), and other reasons (7%). The rationale was unclear in 6% of cases (Figure 2).

Other reasons include: 2 cases of acute pancreatitis,1 case of osteogenesis imperfecta, 2 cases of umbilical cord prolapse, 1 case of maternal obesity, 1 case of cerebral palsy, 1 case of maternal rejection of spinal anesthesia.

### Baseline maternal and neonatal characteristics

No significant differences were observed between the two groups regarding maternal age, primiparity status, incidence of primary cesarean section, gestational diabetes, and prenatal magnesium sulfate use. However, the GA group exhibited significantly higher maternal BMI (29.8 ± 0.5vs 28.5 ± 0.1, *p* = 0.024), prevalence of hypertensive disorders of pregnancy (71/124 [57.2%] vs. 352/905 [38.9%], *p* < 0.001), and frequency of emergency cesarean sections (98/124 [79%] vs. 577/905 [63.8%], *p* = 0.001) compared to the NA group. Conversely, prenatal glucocorticoid use (86/124 [69.3%] vs. 761/905 [84.1%], *p* < 0.001), utilization of assisted fertility techniques (13/124 [10.5%] vs. 168/905 [18.5%], *p* = 0.028), and incidence of multiple births (24/124 [19.4%] vs. 324/905 [35.8%], *p* < 0.001) were significantly higher in the NA group compared to the GA group (Table 1).

**TABLE 1:** Baseline and demographic characteristics of mothers and neonates.

	GA (*n* = 124)	NA (*n* = 905)	Chi-square value	*p-*value
Maternal and perinatal variables				
Maternal age, yr	2 (29–36)	1 (29–35)	1.305^#^	0.192
BMI at delivery, kg/m^2^	29.8 ± 0.5	28.5 ± 0.1	3.009^&^	0.024
Primiparity, n (%)	80 (64.5)	56 (62.2)	0.247	0.619
Primary CS, n (%)	79 (63.7)	591 (65.)	0.122	0.727
Multiple gestation, n (%)	24 (19.4)	24 (5.8)	13.179	<0.001
Assisted Reproductive Technology, n (%)	1 (10.5)	168 (18.5)	4.799	0.028
Emergency CS, n (%)	98 (79.0)	577 (63.8)	11.137	0.001
Hypertensive disease of pregnancy^a^, n (%)	71 (57.2)	352 (38.9)	15.190	<0.001
Diabetes^b^, n (%)	27 (21.8)	169 (18.5)	0.410	0.410
Glucocorticoids use before delivery, n (%)	86 (69.3)	761 (84.1)	16.261	<0.001
Magnesium sulfate use before delivery, n (%)	63 (50.8)	475 (52.5)	0.123	0.725
Neonatal variables				
Birthweight, g	1139.8 ± 32.2	1212.1 ± 11.1	2.235^&^	0.026
Gestational age, week	29.7 (28.1–30.8)	29.7 (28.4–31.1)	1.113^#^	0.266
Small for gestation, n (%)	12 (9.6)	92 (10.1)	0.029	0.851
Male, n (%)	62 (50.0)	501 (55.)	1.323	0.25

#: Z value, &: t value.

^a^ Hypertensive disease of pregnancy: including Chronic hypertension, Gestational hypertension, Preeclampsia/eclampsia, Superimposed.

^b^ Diabetes: including Prepregnancy diabetes, Gestational diabetes, impaired glucose tolerance. GA: general anesthesia, NA: neuraxial anesthesia, BMI: body mass index, CS: cesarean section.

Regarding neonatal baseline characteristics (Table 1), no significant differences were found in gestational age at birth, sex distribution, and the incidence of being small for gestational age between the two groups. However, birth weight(g) was significantly lower in the GA group compared to the NA group (1139.8 ± 32.2vs 1212.1 ± 11.1, *p* = 0.026).

## Adverse outcomes of neonates delivered under GA and NA

In our analysis of neonatal outcomes between the two groups, VPIs in the GA group demonstrated a higher incidence of low Apgar scores (≤7) at 1 minute (102/124 [82.2%] vs. 404/905 [44.6%], *p* < 0.001) and 5 minutes post-delivery (83/124 [66.9%] vs. 290/905 [32.0%], *p* < 0.001) compared to the NA group. Additionally, the necessity for resuscitation through tracheal intubation in the delivery room was more prevalent in the GA group (40/124 [32.2%] vs. 110/905 [12.2%], *p* < 0.001). SNI was higher in the GA group compared to the NA group (18/124 [14.5%] vs. 45/905 [5.0%], *p* < 0.001). No significant differences were observed between the two groups in other outcomes, including SNAP-II, mortality within 7 days after birth and ultimately, and the complications of moderate or severe BPD, NEC of stage 2 or higher, and ROP of Grade 3 or higher, as detailed in Table 2.

**TABLE 2:** Adverse outcomes of neonates delivered under GA and NA.

	GA (*n* = 124)	NA (*n* = 905)	Chi-square value	*p-*value
SNAP-II	20 (14–53)	17 (9–53)	1.307^#^	0.140
Low Apgar score at 1 min (≤7), n (%)	102 (82.2)	404 (44.6)	61.941	<0.001
Low Apgar score at 5 min (≤7), n (%)	8 (66.9)	290 (32.0)	57.452	<0.001
Tracheal intubation in delivery room, n (%)	40 (32.2)	110 (12.2)	35.395	<0.001
Death within 7 days, n (%)	7 (5.6)	59 (6.5)	0.139	0.699
Death, n (%)	15 (12.1)	118 (13.0)	0.086	0.753
SNI, n (%)	18 (14.5)	45 (5.0)	17.282	<0.001
Moderate or severe BPD, n (%)	12 (9.7)	758.)	0.272	0.615
NEC ≥ stage 2, n (%)	(2.4)	24 (2.7)	0.023	0.872
Grade ≥3 ROP, n (%)	2 (1.6)	12 (1.3)	0.067	0.796

#: Z value.

GA: general anesthesia; NA: neuraxial anesthesia; SNAP-II: score for neonatal acute physiology II; SNI: severe neurological injury; BPD: bronchopulmonary dysplasia; NEC: necrotizing enterocolitis; ROP: retinopathy of prematurity.

### Association between general anesthesia and adverse outcomes of neonates

The odds of adverse outcomes in VPIs delivered under GA were higher than those under NA, including low Apgar score (≤7) at one and 5 minutes after delivery, as well as SNI and resuscitation by tracheal intubation in delivery room. Adjusted for maternal BMI, multiple gestation, assisted reproductive technology, emergency cesarean, hypertensive disease of pregnancy, glucocorticoids use before delivery and neonatal birthweight, multivariate logistic regression analysis showed that exposure to general anesthesia during cesarean section significantly added the odds of low Apgar score (≤7) at 1 minute (OR adj 6.321; 95% CI 3.729–10.714)and 5 minute (OR adj 4.535; 95% CI 2.975–6.913), SNI (OR adj 3.019; 95% CI 1.615–5.643), and tracheal intubation in delivery room (OR adj 3.133; 95%CI 1.939–5.061) (Table 3).

**TABLE 3:** Multivariate analysis on associations between general anesthesia and adverse outcomes of neonates.

	OR adj (95% CI)	*p*-Value
SNAP-II^a^	0.033 (-2.279-7.255) ^a^	0.306
1-min Apgar score ≤7, n (%)	6.321 (3.729–10.714)	<0.001
5-min Apgar score ≤7, n (%)	4.535 (2.975–6.913)	<0.001
Tracheal intubation in delivery room, n (%)	3.133 (1.939–5.061)	<0.001
Death within 7 days, n (%)	0.767 (0.330–1.785)	0.538
Death, n (%)	0.821 (0.446–1.514)	0.529
SNI, n (%)	3.019 (1.615–5.643)	0.001
Moderate or severe BPD, n (%)	1.049 (0.527–2.085)	0.892
NEC ≥ stage 2, n (%)	1.122 (0.317–3.964)	0.859
Grade ≥3 ROP, n (%)	0.986 (0.205–4.750)	0.986

^a^ The linear regression of the continuous variable is β.

SNAP-II: score for neonatal acute physiology II; SNI: severe neurological injury; BPD: bronchopulmonary dysplasia; NEC: necrotizing enterocolitis; ROP: retinopathy of prematurity. Adjusted for maternal BMI, multiple gestation, assisted reproductive technology, emergency cesarean, hypertensive disease of pregnancy, glucocorticoids use before delivery and neonatal birthweight.

### Association between exposure time to GA and adverse outcomes

124 neonates delivered under GA were divided into four subgroups according to the interval time from skin incision to fetal delivery in ascending order, with 31 cases in each subgroup (Q1∼Q4). The medians and interquartile ranges of the four subgroups are Q1 [2 (2–3)], Q2 [5 (4–5)], Q3 [6 (6–7.5) and Q4 [13 (10–16.5)] respectively (Unit: minute). Chi-square test and Boferroni method were used to determine whether there were statistical differences in variables between multi-subgroups. As shown in Table 4, on the basis of no statistically significant difference in baseline data for neonatal data (birthweight, gestational age, small for gestation, sex), the proportion of neonates with 5-min Apgar score less than or equal to 7 points increased with the extension of the interval time, and the difference in Q4 subgroup was statistically significant (*p* < 0.01). While incidence of low 1-min Apgar score (≦7), SNI and tracheal intubation in delivery room did not differ from every group (Table 4).

**TABLE 4:** Association between adverse outcomes and time from skin incision to delivery.

	Q1 (*n* = 31)	Q2 (*n* = 31)	Q3 (*n* = 31)	Q4 (*n* = 31)	*p*-Value
Birthweight, g	1156.1 ± 54.2	1068.7 ± 49.0	1141.3 ± 63.4	1192.9 ± 86.0	0.594
Gestational age, week	30.1 (29.0, 31.3)	29.4 (28.1, 30.6)	29.4 (27.9, 30.4)	29.9 (26.7, 31.3)	0.585
Small for gestation, n (%)	1 (3.2)	3 (9.7)	3 (9.7)	5 (16.1)	0.443
Male, n (%)	13 (41.9)	20 (64.5)	13 (41.9)	15 (48.4)	0.325
1- min Apgar score ≤7, n (%)	22 (71.0)	25 (80.6)	26 (83.9)	29 (93.5)	0.137
5-min Apgar score ≤7, n (%)	14 (45.2)	18 (58.1)	24 (77.4)	27 (87.1) *	0.002
SNI, n (%)	2 (6.5)	8 (25.8)	5 (16.1)	3 (9.7)	0.141
Tracheal intubation in delivery room, n (%)	9 (29.0)	7 (22.6)	13 (41.9)	11 (35.5)	0.399

*p < 0.01 VS Q1.

## Discussion

In this observational study, we observed an association between adverse neonatal outcomes and the use of GA in VPIs delivered by cesarean section. Specifically, GA was linked to a higher frequency of perinatal adverse outcomes, including low Apgar scores at one and 5 minutes, the need for tracheal intubation at birth, and SNI, compared to neonates delivered under NA. This association remained significant even after adjusting for potential confounding clinical factors. Additionally, a prolonged duration of GA exposure was correlated with low 5-min Apgar score in neonates.

Data from a 2015 multi-center United States survey revealed that 17.6% of women underwent cesarean sections between 24 + 0 and 36+6 weeks of gestation via GA (Butwick et al., 2015). The proportion of GA in our study was marginally lower, potentially attributable to variations in the gestational ages of the preterm infants examined and advancements in anesthesia techniques and perinatal management in recent years (Bollag et al., 2021).

Cesarean sections can be executed under NA, encompassing spinal anesthesia and epidural anesthesia, or under GA. The selection of anesthesia mode is typically influenced by clinical indications, the anesthesiologist’s experience, and maternal preferences. NA provides the advantage of maternal consciousness during cesarean delivery and minimizes anesthetic exposure to the neonate. Additionally, NA reduces the risks of maternal aspiration and difficult airway management, commonly associated with GA (Mushambi et al., 2015). NA remains the preferred and gold standard mode of anesthesia for cesarean sections. However, specific conditions necessitate considering GA as a secondary option. These include infection at the needle insertion site, significant coagulopathy, hypovolemic shock, increased intracranial pressure due to space-occupying lesions, and scenarios where provider expertise in NA is inadequate or other critical conditions that endanger maternal-fetal life and require immediate termination (Afolabi and Lesi, 2012). Our findings indicate that maternal thrombocytopenia or coagulation disorders, and the use of antiplatelet/anticoagulation medications, are primary factors influencing the choice of GA. Pre-eclampsia continues to be a leading cause of maternal and neonatal morbidities, particularly in developing countries. The definitive treatment for pre-eclampsia involves timely termination of pregnancy, which consequently leads to a higher incidence of cesarean sections among VPIs in pre-eclamptic women (Liu et al., 2008; Nankali et al., 2012). Thrombocytopenia and coagulation disturbances, key characteristics of HELLP syndrome, often contraindicate the use of NA (Adorno et al., 2022; del-Rio-Vellosillo and Garcia-Medina, 2015). This may elucidate our finding that mothers with HELLP syndrome exhibited a higher likelihood of undergoing GA in our study. Additionally, we observed a significantly higher proportion of VPIs from emergency cesarean sections in the GA group. In scenarios such as placental abruption, placenta previa with massive bleeding, umbilical cord prolapse, or imminent fetal heart rate loss, both obstetricians and patients often favor GA due to its rapid induction and reduced cardiovascular instability (Guglielminotti et al., 2019; Ikeda et al., 2020). Previous research identified obesity as a risk factor for the selection of GA during cesarean sections (Butwick et al., 2015). Our current study corroborates this finding, noting a significantly higher maternal BMI in the GA group compared to the NA group, aligning with their results.

The choice of anesthesia during cesarean section can significantly impact neonatal outcomes (Saygı et al., 2015). Research exploring the relationship between anesthesia mode during cesarean delivery and outcomes in preterm infants has yielded varying results. One study indicated that mortality rates in preterm infants (<33 weeks’ gestational age) born to women receiving SA were higher compared to those born under GA. Conversely, another cohort study focusing on preterm infants ≤32 weeks’ gestational age found that neonates born to women receiving EA had higher Apgar scores than those from mothers who received GA (Rolbin et al., 1994; Laudenbach et al., 2009). The Apgar scoring system, a traditional and practical method for immediate neonatal assessment post-delivery, is crucial for identifying infants in need of resuscitation and predicting perinatal survival (Casey et al., 2001). In our study, the incidence of low 1-min and 5-min Apgar scores among VPIs was significantly higher, and a greater number of VPIs in the GA group required resuscitation with tracheal intubation in the delivery room. These findings indirectly reaffirm the importance of this traditional evaluation method.

Research indicated that the impact of drugs related to GA on the developing brain was mediated through both morphological alterations and molecular mechanisms (Vutskits and Xie, 2016). Groundbreaking studies, particularly within the scope of fetal alcohol syndrome, had elucidated that exposure to substances functioning as pharmacological antagonists of the NMDA-type glutamate receptor (NMDAR) or as positive allosteric modulators of the type A GABA receptor (GABAAR)-mediated neurotransmission, precipitated rapid cellular demise during specific developmental periods in the immature rodent brain, contingent on the brain region (Ikonomidou, C. et al., 1999; Ikonomidou, C. et al., 2000). Preliminary investigations had uncovered GA prompt region-specific temporal alterations in the expression of brain-derived neurotrophic factor (BDNF), with the subsequent modifications in the activity of downstream signaling pathways posited to trigger the initiation of apoptotic sequences (Yon, J. H., et al., 2005; Popic, J. et al., 2012). In recent years, the potential neurotoxic effects of GA on the developing brain have garnered increased attention. Animal studies suggest that exposure to GA agents *in utero* can induce apoptosis and developmental neural degeneration in the fetal brain (Palanisamy, 2012). Notably, the most critical period for anesthetic-induced neurodegeneration is approximated to be around the 26th week of gestation in humans (Clancy et al., 2007). Given the heightened susceptibility of VPIs to maternally administered drugs, due to immature enzyme systems, an incomplete blood-brain barrier, and limited serum protein availability for drug binding, it is crucial to assess both early neonatal outcomes and the long-term effects of anesthetic exposure. Various anesthetic agents can have a broad spectrum of impacts on newborns. Consequently, our study also examined the well-defined adverse outcomes associated with long-term survival and quality of life in VPIs, including SNI, BPD, NEC, and ROP. IVH is a prevalent type of brain injury in preterm infants, often leading to complications such as PVL (Ballabh, 2010; Su et al., 2016). Studies indicate an increased incidence of severe IVH with decreasing gestational age and birth weight (Zhao et al., 2022). Additionally, low Apgar scores are well-established risk factors for IVH (Coskun et al., 2017; Zea-Vera et al., 2019). This could explain the higher prevalence of SNI observed in VPIs from the GA group compared to those from the NA group in our study.

Few studies have explored the association between the interval from anesthesia induction to birth and perinatal outcomes during GA, yielding mixed results. Kate Swanson’s research indicated that a longer interval time (exceeding 4 min) from the initiation of general endotracheal anesthesia to cesarean delivery correlates with a higher frequency of perinatal complications. In bivariable analysis, a 5-min Apgar score of less than 7 was more prevalent in groups with an interval time exceeding 4 min. However, this association did not remain significant in multivariable analysis (Swanson et al., 2021). Hu L and colleagues reported that extending the interval from anesthesia induction to delivery, within certain limits, does not significantly effect fetal outcomes in full-term cesarean deliveries (Hu et al., 2017). However, these studies focused solely on full-term deliveries. In contrast, our current study observed that a longer interval from skin incision to delivery during GA was associated with lower 5-min Apgar scores in preterm deliveries. This finding suggests that, in addition to large molecule drugs like non-depolarizing muscle relaxants, other GA-related drugs may also cross the placental barrier and effect the fetus. Common obstetric general anesthesia agents, such as propofol and ketamine, are associated with increased risks of tachycardia, hypertension, and respiratory adverse events in infants, including cough and laryngospasm (Hayes et al., 2020). Recent studies suggest the feasibility of using opioids at the time of maternal anesthesia induction (Caissie et al., 2022). However, concerns about neonatal respiratory depression risk often leads anesthesiologists to avoid these drugs during induction. Prolonged skin incision-to-delivery intervals under GA may result in increased fetal exposure to anesthetic drugs, potentially elevating the risk of fetal respiratory depression. Minimizing exposure duration to GA could therefore offer clinical benefits for VPIs.

This study has several limitations. First, we were unable to evaluate certain factors, notably the interval from the decision to perform a cesarean section to the delivery of the neonate, a critical determinant of neonatal outcomes. This is partly because prolonged delivery times in emergency situations often involve logistical challenges, such as transporting the patient to the operating room (Wong et al., 2017; Temesgen et al., 2020). Second, despite our efforts to adjust for relevant confounders, residual confounding biases may still exist. This limitation stems from the retrospective nature of our study, wherein data were occasionally recorded inconsistently or inaccurately in medical records.

In conclusion, our study suggests that for VPIs delivered via cesarean section, GA is associated with lower Apgar scores, increased need for tracheal intubation resuscitation, and adverse neurological outcomes. Prolonged exposure to GA may contribute to neonatal postnatal depression. While these findings do not negate the utility of GA in cesarean sections for VPIs, they underscore the importance of specific precautions. These include minimizing the duration of fetal delivery and ensuring adequate preparation for neonatal resuscitation when GA is selected for cesarean sections in VPIs.

## Data Availability

The datasets generated and analyzed in the current study are not readily publicly available because the data is collected from Shandong Neonatal Network (SNN), and our relevant research has not been published. Requests to access the datasets could be directed to the corresponding author (YY, alice20402@126.com).
